# Streamlined procedure for gene knockouts using all-in-one adenoviral CRISPR-Cas9

**DOI:** 10.1038/s41598-018-36736-y

**Published:** 2019-01-22

**Authors:** Yuan-Hu Jin, Hyunjeong Joo, Kwangjun Lee, Hyeongseok Kim, Ruth Didier, Young Yang, Heungsop Shin, Choogon Lee

**Affiliations:** 1 Department of Biomedical Sciences, Program in Neuroscience, College of Medicine, Florida State University, 1115 West Call Street, Tallahassee, FL 32306 USA; 2 Division of Biological Sciences, Department of Life Systems, Sookmyung Women’s University, Seoul, Republic of Korea; 3 Department of Chemical Engineering and Biotechnology, Korea Polytechnic University, Siheung, Republic of Korea

## Abstract

CRISPR-Cas9 is a powerful gene editing technique that can induce mutations in a target gene of interest in almost any mammalian cell line. However, its practicality can be limited if target cell lines are difficult to transfect and do not proliferate. In the current study, we have developed a streamlined approach for CRISPR-based gene knockouts with three key advantages, which allows phenotypic assay of gene knockouts without clonal selection and expansion. First, it integrates into a single, all-in-one vector transgenes for Cas9, sgRNA, and a fluorescence marker. Second, we used the Gateway system to rapidly clone specific sgRNAs into the all-in-one vector through PCR and *in vitro* recombination, without conventional enzyme digestion and ligation. Third, it uses adenovirus for the capacity to package the all-in-one vector, and for its high efficiency of transduction. We tested the all-in-one adenoviral CRISPR-Cas9 in a circadian clock model cell line U2OS, and demonstrated that essential clock genes such as *Bmal1* and *Per1* were knocked out so efficiently that functional assays could be performed from the heterogenic population without any clonal selection and expansion. This streamlined approach may prove invaluable for rapid functional assays of candidate genes in diverse biological pathways, including the circadian clock.

## Introduction

The prokaryotic defense system CRISPR-Cas was discovered as an adaptive immunity against foreign DNA molecules and converted to a revolutionary genome editing tool^1–3^. Unlike the siRNA approach, CRISPR-Cas can permanently alter the function of genes by mutating them. CRISPR-Cas9 derived from *Streptococcus pyogenes* is the most widely used system because of its ease of use and effectiveness^4^. The system requires introducing two components: the nuclease Cas9 and a single-guide RNA (sgRNA) which targets the nuclease to a specific genomic locus based on base pairing at a 20-nt target sequence. The target sequence must be followed immediately by the motif 5′-NGG-3′, which is called PAM (protospacer adjacent motif)^4^. The sgRNA-guided Cas9 generates a double strand break (DSB) in the target site, which may modify the genome in two different ways. The first is random insertion/deletion (indel) mutagenesis which occurs when the DSB is repaired by the error-prone non-homologous end-joining (NHEJ) pathway, often resulting in a knockout or loss-of-function mutation^4^. The second is precise allele editing through the high-fidelity homology-directed repair (HDR) pathway, which may occur in a small percentage of the cells (<5%)^5,6^. More definitive than siRNA, more precise than older transgene insertion methods, and more efficient than older homologous recombination methods^7,8^, CRISPR has become one of the most widely used methods to alter a gene to interrogate its function or for medical applications^9,10^.

However, CRISPR has limitations. Transfection of the Cas9 and sgRNA in plasmids typically only succeeds for a minority of cells, and in certain cell lines which do not proliferate or are not easily transfected with plasmids, clonal selection and expansion from the transfected heterogenic population may not be practical or possible. Various CRISPR viral vectors have been employed to try to overcome these problems^11–16^, but clonal selection and expansion would be still required unless the transduction efficiency and expression of the viral vectors are very high. Among the most widely used viral vectors, lentivirus and adeno-associated virus (AAV) are preferred for human gene therapy applications, but adenovirus (AV) has important advantages as a research tool^17,18^. AV has the highest transduction efficiency in diverse target cells^16,19,20^; unlike lentivirus, its genome does not integrate into the host genome, thus reducing off-target mutations; and AV has a substantially higher genome capacity than AAV^21^. We have used this capacity to co-package three CRISPR-Cas9 components along with their promoters: Cas9, sgRNA and a fluorescent protein to aid in sorting for transduced cells. A fluorescent protein such as GFP or mCherry is also used to monitor active viral production in packaging cell lines such as 293 A and 911^21^. When active adenoviral particles are made and released from the cells, they infect neighboring cells forming comet-like plaques which can be monitored under fluorescent microscopy. Others have previously demonstrated the feasibility of using AV to deliver Cas9 and sgRNA into diverse target cells, including primary and immortalized cells *in vitro*, and *in vivo*^15,16,18^. Most have (like us) used a replication-incompetent AV5 vector lacking E1 and E3; one group used a new AV vector devoid of all viral genes^14^, but with the trade-off of a more cumbersome production process and less infectable AV. In this study, we demonstrate the efficacy and versatility of an all-in-one adenovirus CRISPR-Cas9 system to target a specific biological pathway, the circadian clock.

The circadian clock is a molecular circuit that controls daily rhythms in physiology and behavior, such as metabolic oscillations and wake-sleep cycles^22–24^. Adverse consequences from having a faulty clock include compromised sleep quality, poor work performance, and increased risk for accidents in the short-term, as well as metabolic diseases and cancer in the long-term. Because the circadian clock is cell autonomous, and rhythms can be measured in cells in real-time using a luciferase reporter under control of a clock promoter, clock mechanisms can be studied in cell culture models such as mouse embryonic fibroblasts (MEFs) or human cell line U2OS^25–27^. Clock gene regulation, protein rhythms, and protein-protein interactions in cultured cells are comparable to those in *in vivo* tissues^28,29^. The molecular backbone of the circadian clock is a transcriptional negative feedback loop with interacting positive and negative elements^30,31^. CLOCK and BMAL1 are the positive elements, activating transcription of many downstream genes, including behavior-regulating genes and the main negative elements, *Period* (*Per*, including *Per1* and *Per2*) and *Cryptochrome* (*Cry*, including *Cry1* and *Cry2*), whose products form an auto-inhibitory complex^30,31^. Unlike other core clock genes, *Bmal1* has no redundant paralog. Disruption of *Bmal1* results in complete arrhythmicity while disruption of a single *Cry* or *Per* family member produces subtle circadian phenotypes^32–34^.

In the current study, we developed an all-in-one adenoviral vector where sgRNA can be easily cloned by the Gateway cloning system without labor-intensive conventional cloning and selection. Our vector achieves packaging of sgRNA, the SpCas9 transgene, and a fluorescent protein transgene into the same viral particle. Since indel efficiency of CRISPR-Cas9 is very high, up to ~100%^35^, and adenovirus-mediated transduction is ~100% efficient and not cytotoxic, clonal selection and expansion may not be necessary to study the phenotype of knockouts if targeting is properly designed. We showed that a complete disruption of the circadian clock can be achieved in cell culture without clonal selection and expansion by targeting a splicing site in the essential clock gene *Bmal1* using the all-in-one adenoviral CRISPR-Cas9 system. We believe interrogation of gene function can be performed in diverse cell lines in a rapid manner by using our all-in-one adenoviral CRISPR-Cas9 system since it does not involve conventional cloning, clonal selection and expansion.

## Results

### Generation of all-in-one CRISPR-Cas9 vector using the Gateway cloning technology

To improve upon the conventional procedure of cloning sgRNA into plasmids using enzyme digestion and ligation^4^, we adopted the Gateway cloning technology. Gateway technology was developed by Invitrogen to transfer DNA fragments between plasmids using the bacteriophage lambda site-specific recombination system^36^. Briefly, a DNA fragment of interest is first cloned into an Entry plasmid where the DNA fragment becomes flanked by specific recombination sequences called attL1 and attL2. The DNA fragment in the Entry plasmid can then be transferred to any Destination vector by LR recombinase. Since only positive recombinants are selected, the subcloning procedure is highly efficient and rapid.

**Figure 1:**
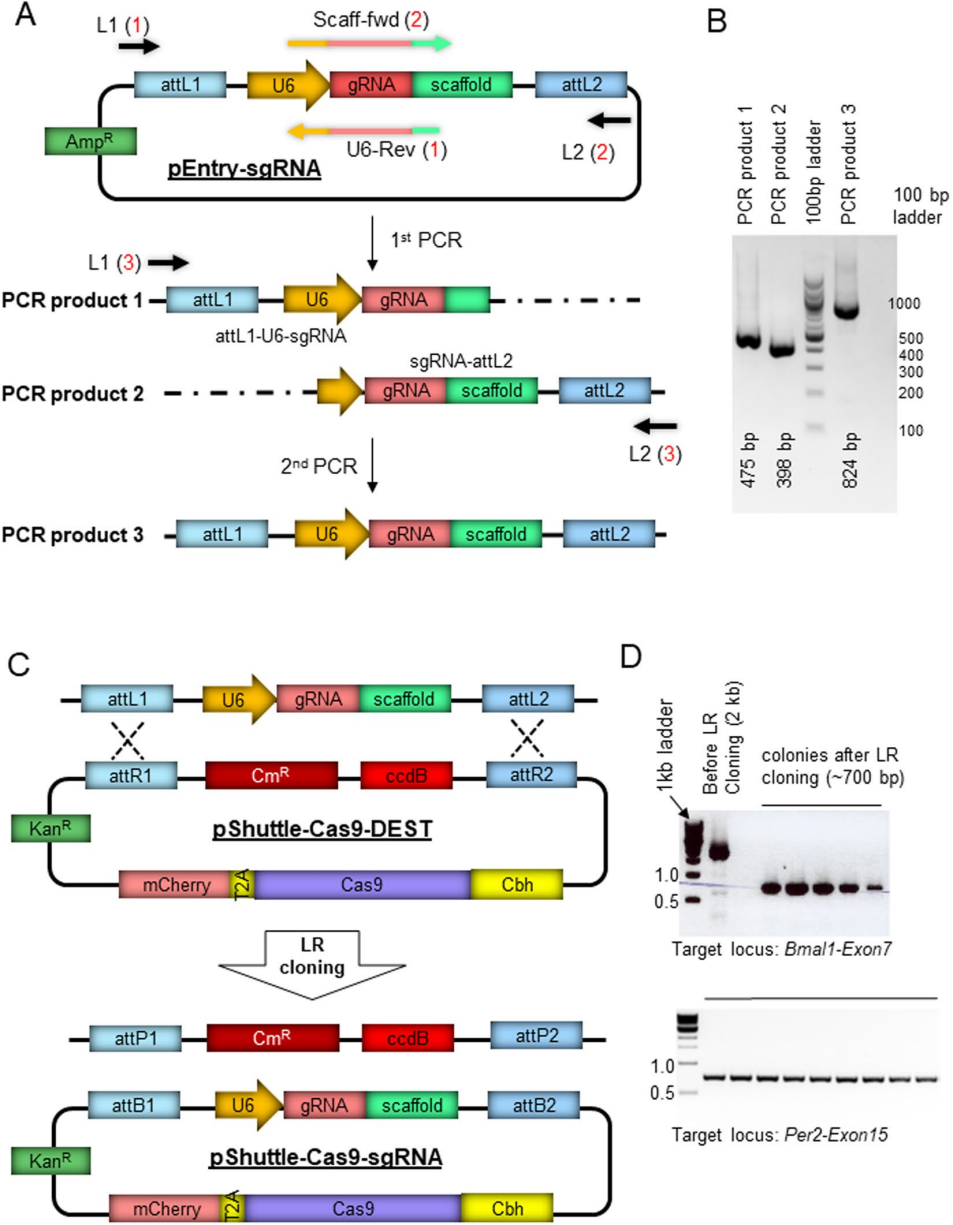
Generation of an all-in-one Cas9-mCherry-sgRNA vector using the Gateway system. (**A**) A Gateway entry plasmid was generated to clone a specific sgRNA into a PCR amplicon flanked by attL1 and attL2. PCR from the Entry vector using two sets of primers with specific 20 nt gRNA sequence (reaction 1 and 2) produces two fragments that partially overlap and can be combined by an overlap extension PCR (reaction 3). Multiplexing is possible by using multiple sets of Scaff-fwd and U6-Rev primers. (**B**) PCR products of two individual fragments and a full-length L1-U6-sgRNA-L2 are shown. (**C**) The final PCR amplicons can be combined with the adenoviral shuttle vector with R1-ccdB-R2 Destination sequence or used separately. pShuttle-Cas9-DEST was generated by cloning Cas9-mCherry and R1-R2 into pShuttle. (**D**) Gateway LR cloning produces 100% positive clones. Since the background (before recombination) clones cannot grow in ccdB-incompatible bacterial cell lines such as DH5a, all the transformed colonies with the LR reaction mixture contain the recombinant without ccdB, but with sgRNA. Two independent experiments are shown.

To clone specific sgRNA into an all-in-one Destination vector, we first generated a specific linear sgRNA transgene flanked by attL1 and attL2 through two rounds of PCR using a Gateway Entry template (pEntry-sgRNA) (Fig. 1A,B). Briefly, PCR from the Entry vector using two sets of primers with specific 20 nt gRNA sequence produces two fragments that partially overlap (PCR products 1 and 2, Fig. 1A), and can be combined by an overlap extension PCR to produce a final linear sgRNA transgene with U6 promoter and poly-A sequence along with attL1 and attL2 (product 3, Fig. 1A). The final, full-length PCR amplicon was used as a Gateway sgRNA Entry vector to deliver U6 promoter-sgRNA-poly A into a Gateway Destination vector which expresses Cas9-mCherry fusion protein under a *Cbh* promoter (pShuttle-Cas9-DEST) (Fig. 1C). Recombination between the linear PCR amplicon and the pShuttle-Cas9-DEST was mediated *in vitro* by LR clonase (Fig. 1C). It has been shown that LR recombination occurs efficiently regardless of the topology of the DNA, whether it is linear (e.g., PCR products) or circular. As shown in Fig. 1D, all the bacterial transformants harbored the positive recombinant because the non-recombinant plasmid (pShuttle-Cas9-DEST) is toxic (due to ccdB) to standard competent cells such as DH5a.

### Functional analysis of the all-in-one vector

Before packaging the vector into adenovirus, we first tested the functionality of the all-in-one vector construct itself. We used it to edit essential clock genes—whose null phenotypes were well characterized—by transfecting it into a human cell line, U2OS, expressing a rapidly degraded luciferase (dLuc) under a *Bmal1* promoter^27^. This cell line is widely used as a model system to study clock mechanisms, and bioluminescence signal from *Bmal1*-dLuc (or a similar reporter) is widely used as a real-time clock reporter since it faithfully reports the activity of the endogenous clock mechanism.

**Figure 2:**
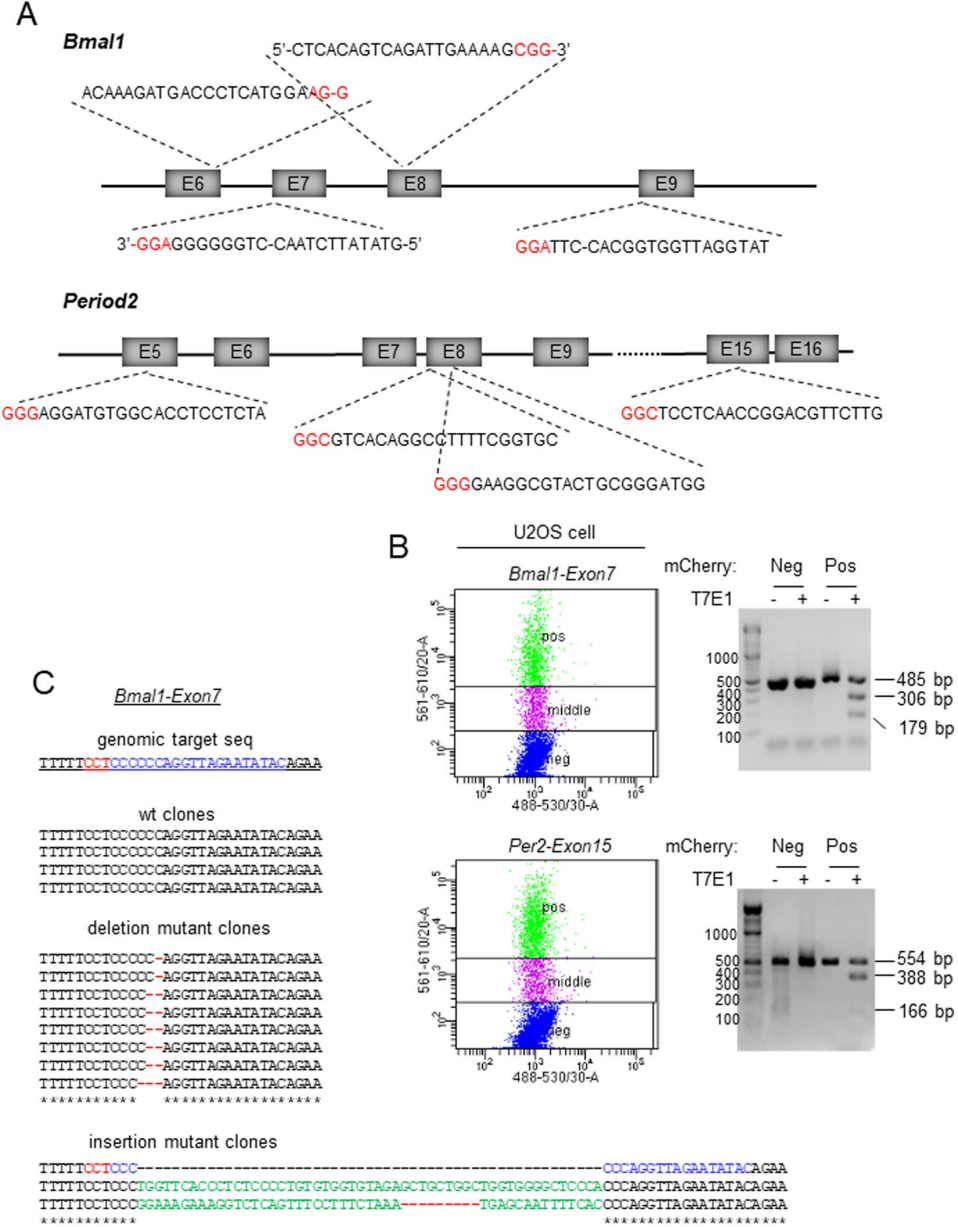
Efficient targeting of *Bmal1* and *Per2* genes in U2OS cells by the all-in-one CRISPR-Cas9 vector. (**A**) Four exons were targeted in each gene based on their score in a predictive software tool. The PAM sequences are indicated in red. Note that target sites for *Bmal1* exon 6, 7 and 9 are close to splicing sites indicated by dashes within the sequence. (**B**) mCherry-expressing cells (Pos) were selected by FACS and subjected to T7E1 assay to assess the efficiency of indels. Control cells (Neg) were from the same FACS sorting. Cells with low mCherry signal (middle) were discarded. All of the eight samples showed similarly efficient indels, proportional to transfection efficiency. Two representative samples are shown. (**C**) Targeting of the clock genes by the all-in-one vectors produces diverse indels including frame-shifting mutations. PCR amplicons from the genomic targets of *Bmal1* exon 7 and *Per2* exon 15 were sequenced (see Supplementary Fig. 1 for *Per2* exon 15). 15–30% of clones were wt. Deletions and insertions are indicated by lines and green characters, respectively.

One of the genes we targeted was *Bmal1*. In the mammalian system, *Bmal1* is the only essential clock gene without redundancy^32^. Thus, if CRISPR-Cas9 generates frame-shifting mutations (via NHEJ) in early exons of both alleles of *Bmal1*, the C-terminal transcriptional activation domain of BMAL1 will be deleted, resulting in circadian arrhythmicity^37^. Aided by an in silico design tool (http://chopchop.cbu.uib.no), we identified sgRNA sequences targeting four exons of *Bmal1* (exons 6, 7, 8 and 9) based on the 20 nt sequence immediately preceding SpCas9 PAM sequences in those exons (Fig. 2A). We also targeted another critical clock gene, human *Period2*, to demonstrate that efficiency of our all-in-one vector is not limited to a specific gene. Four exons of *Per2* were targeted (Fig. 2A).

U2OS cells transfected with each of the all-in-one plasmids were FACS-sorted based on mCherry fluorescence (Fig. 2B). When target PCR amplicons from genomic DNA extracted from the positive cells were subjected to T7E1 digestion, indels were detected in all of the samples (Fig. 2B). When the PCR amplicons from *Bmal1-exon 7 and Per2-exon15-*targeted samples were sequenced, wild-type (wt) clones were detected as well as indels (Fig. 2C and Supplementary Fig. 1). We believe this is due to incomplete trypsinization of transfected cells, which resulted in inclusion of wt cells among the FACS-sorted mCherry cells, rather than inactivity of Cas9-sgRNA complex in certain cells, because some of the FACS-sorted cells did not express mCherry when they were inspected under fluorescence microscopy.

**Figure 3:**
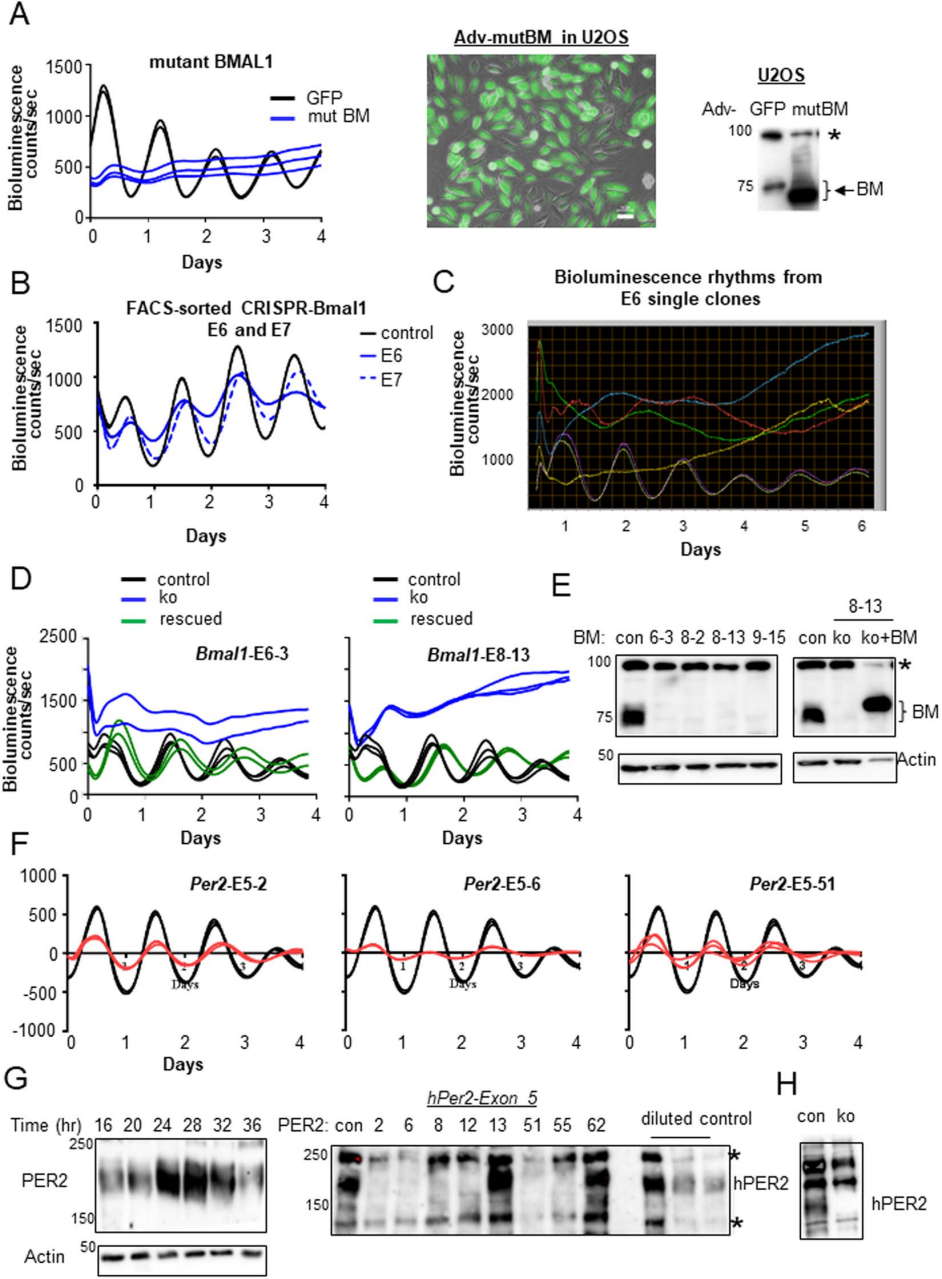
Efficient generation of *Bmal1* and *Per2* knockout clones in U2OS cells by the all-in-one vector. (**A**) Function of *Bmal1* is essential for the clock in U2OS cells. *Bmal1* promoter driven Luciferase rhythms are completely disrupted by overexpressing a mutant BMAL1 lacking the DNA-binding domain. Transduction efficiency by the adenovirus was ~100% (see Supplementary Fig. 5) as shown in the merged image between bright field and GFP. Expression levels of the transgene were ~10-fold higher than those of endogenous *Bmal1* (right panel). *Indicates a nonspecific band. Note that the mutant BMAL1 is smaller than the endogenous one. The scale bar represents 50 μm. (**B**) Circadian rhythms are still observed when *Bmal1* exon 6 and 7 are targeted by transfection of the all-in-one CRISPR-Cas9 vector, and positive cells were selected by FACS and subjected to bioluminescence assay. Amplitude was reduced in both cases. (**C**) Arrhythmic clones are easily selected by single cell isolation and expansion from the FACS sorted U2OS cells. Four arrhythmic clones from *Bmal1* exon 6 targeted cells are shown along with two control traces. Raw bioluminescence rhythms are shown. Efficiency of knockouts (number of arrhythmic clones/total number) for E6 = 23/37 (62%); for E8 = 9/17 (53%); for E9 = 5/16 (31%). (**D**) Arrhythmicity is not due to off-target effect of the all-in-one CRISPR-Cas9. All arrhythmic clones tested were rescued by expressing wt BMAL1 protein using the adenoviral vector. Two representative cases are shown. Blue: arrhythmic clone; Green: the arrhythmic clone plus AV-*wtBmal1*; Black: control cell. (**E**) Knockouts were confirmed by immunoblots and sequencing (Supplementary Fig. 2). Representative arrhythmic clones from three different experiments (*Bmal1* exon 6, 8 and 9) are shown. Note that the BMAL1-rescued sample was under-loaded to show the difference in size clearly between endogenous and transgenic BMAL1 due to 3XFlag tag. (**F,G**) Knockout clones for *Per2* were efficiently generated by the all-in-one vector targeting exon 5. Three knockout clones are shown, which was confirmed by immunoblots (**G**) and sequencing (Supplementary Fig. 4). Note that all knockout clones (red) show damped rhythms compared to control cells (black). The same control traces and the same scale for Y-axis were used in all three graphs. The graphs were de-trended to compare amplitudes between control and knockout samples. (**G**) PER2 exhibits robust rhythms in U2OS (left panel). The samples were harvested at the indicated times after 2-hr serum shock. The majority of clones with damped rhythms show absence of PER2 (middle panel). Note that serially diluted control samples (1, 1/2 and 1/3) were loaded next to the candidate samples to show the detection of PER2 at those levels and the corresponding absence in knockout clones. Note that clones 5–13 and 5–62 retain PER2 expression. *Indicates a nonspecific band. (**H**) Knockout was independently confirmed by a novel monoclonal antibody. Probing of time-course samples with the antibody showed similarly robust oscillations (Supplementary Fig. 7).

Functional consequences of these indels in the clock genes were analyzed by measuring bioluminescence rhythms in singly isolated and expanded clones. We have previously shown that overexpression of a truncated mutant BMAL1 lacking the DNA binding domain can completely disrupt clock function in MEFs, and the serotype 5 adenoviral vector can transduce the mutant *Bmal1* gene into MEFs with ~100% efficiency^19,38^. We used the same adenoviral vector to transduce the mutant *Bmal1* into U2OS-*Bmal1-luc* cells to verify that the disruption of BMAL1 function produces a similarly compromised clock phenotype in U2OS cells (Fig. 3A). Indeed, bioluminescence rhythms were completely disrupted in U2OS cells when transduction of adenovirus into the cells is close to 100%. When we transfected the CRISPR all-in-one plasmids into U2OS cells to target *Bmal1* exon 6 or exon 7, FACS-sorted the cells and assessed bioluminescence rhythms without clonal separation and expansion, the cells still showed rhythms although their amplitude was significantly reduced (Fig. 3B). This is most likely due to contamination of wt clones, as suggested by the sequencing results, in addition to potential in-frame mutant clones. To measure efficiency of frame-shifting mutations in all alleles (complete knockouts) in cells by our method, three exons of *Bmal1*, exon 6, 8 and 9 in the cells were targeted, FACS-sorted into single cells and expanded. When these clones were subject to bioluminescence assay, 62, 53 and 31% of clones were arrhythmic upon targeting of exon 6, 8 and 9, respectively (arrhythmic clones from exon 6 are shown in Fig. 3C). The arrhythmicity was not due to off target mutations since arrhythmic clones were rescued by expressing wt BMAL1 in them (Fig. 3D)^38^. Knockouts of *Bmal1* were confirmed by immunoblotting and sequencing (Fig. 3E and Supplementary Fig. 2) in some of the arrhythmic clones. A similarly efficient knockout was observed when *Per2*-exon 5 was targeted (Fig. 3F–H and Supplementary Fig. 3). Consistent with a previous study, deletion of *Per2* in U2OS generated dampened rhythms^39^. Some of the *Per2* mutant clones exhibited dampened rhythms (e.g., #5–13, Supplementary Fig. 4) or altered circadian period (e.g., #5–62, Supplementary Fig. 4), yet retained PER2 expression, suggesting that these clones have non-frame-shifting indels in the target.

### Knockout of a gene in cell culture in one step by adenoviral all-in-one vector

We thus confirmed the functionality of our all-in-one plasmid, but also confirmed the limitations of the typical paradigm of CRISPR-Cas9 mutagenesis^4^, where the inefficiency of transfection requires that cells be sorted or selected based on fluorescence markers (or alternatively, drug selection genes), and then the positive cells are expanded before subjected to functional assays. This approach can be applied to many cell lines, but it cannot be used in many primary cell lines that do not proliferate or cell lines that are very difficult to transfect. Moreover, clonal selection and expansion by FACS or drug selection may be labor-intensive and time-consuming, and demands a certain level of expertise.

**Figure 4:**
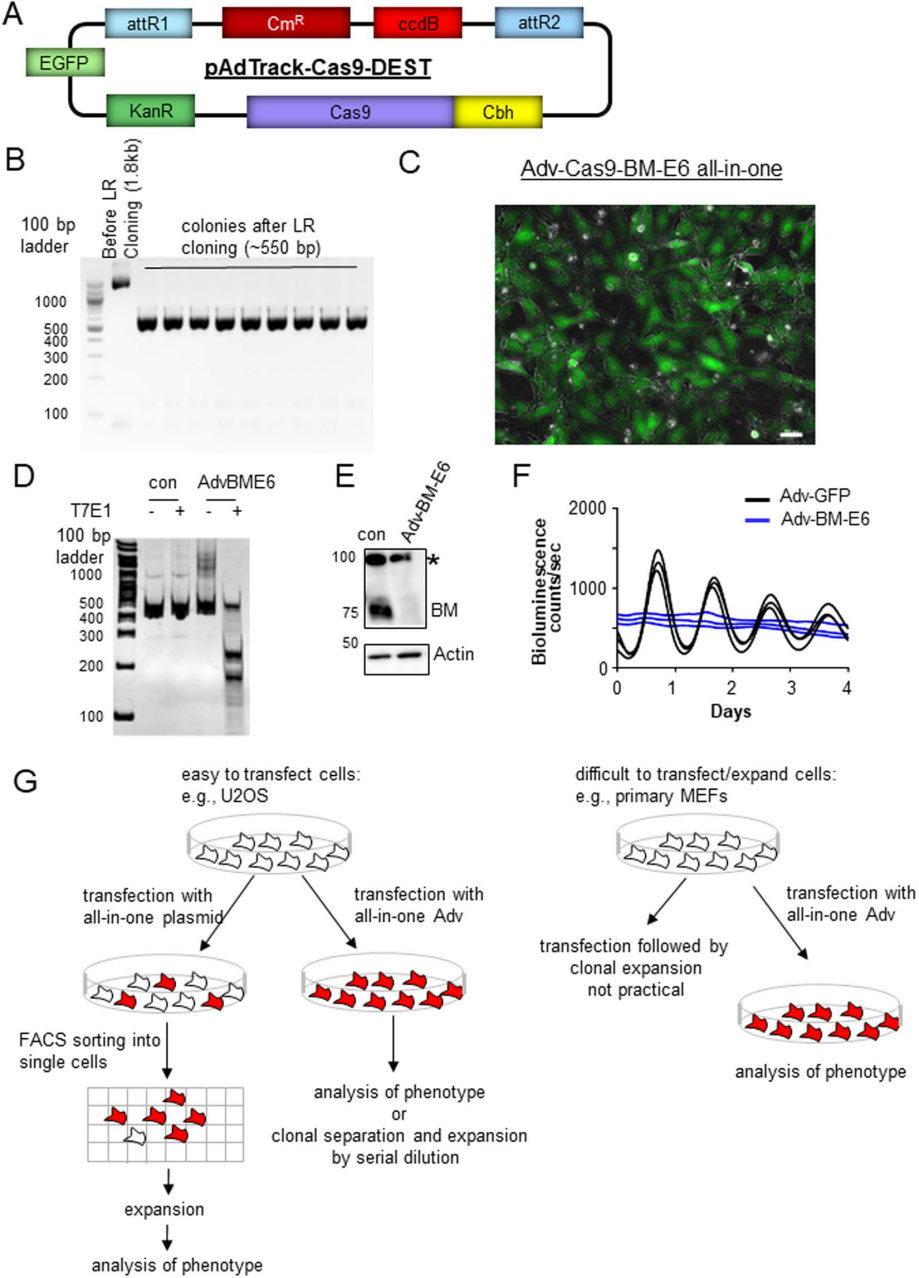
All-in-one adenovirus targeting *Bmal1* exon 6 can generate knockouts in the majority of U2OS cells without FACS sorting. (**A**) Generation of a new adenoviral sgRNA-DEST vector. Cas9 and R1-ccdB-R2 were cloned into the pAdTrack vector. Note that mCherry is deleted and EGFP is expressed by a separate promoter. (**B**) Efficient cloning of sgRNA by LR cloning. The same strategy as shown in Fig. 1 was used to clone specific sgRNA into the all-in-one vector. (**C**) Confirmed packaging and efficient transduction by the all-in-one adenovirus. The all-in-one vector can be effectively packaged into AV and transduced into U2OS cells with >98% efficiency (see Supplementary Fig. 5). To ensure ~100% transduction and high level expression of Cas9:sgRNA complex in the cells, U2OS cells were infected twice at a Multiplicity Of Infection (MOI) of 50. The scale bar represents 50 μm. A representative case with cell counting is shown in Supplementary Fig. 5. (**D**) T7E1 assay shows a high rate of indels (~70%). Although BME6 amplicons before digestion appear larger than their expected size (378 bp) on PAGE, we found that they have the expected size on agarose gels as shown in Supplementary Fig. 8. (**E**) *Bmal1* was knocked out in the majority of the cells based on immunoblotting. *Indicates a nonspecific band. (**F**) U2OS cells transduced with the all-in-one adenovirus show arrhythmicity. (**G**) Streamlined procedure for gene knockout and analysis of knockout phenotype using all-in-one Adv-Cas9-sgRNA-fluorescent marker vector.

The type 5 adenovirus is highly efficient in delivering transgenes to diverse dividing and non-dividing cells^21^. Thus we hypothesized that high-titer infection of all-in-one CRISPR-Cas9 adenovirus into U2OS cells can achieve ~100% transduction efficiency and generate knockouts in the majority of cells, thus making the downstream clonal selection and expansion unnecessary. To test this possibility, we generated a new adenoviral shuttle vector expressing GFP and Cas9 from two different promoters (pAdTrack-Cas9-DEST) (Fig. 4A). The pShuttle-Cas9-sgRNA construct that we had initially developed failed to produce transducible adenoviral particles when combined with the adenovirus backbone vector pAdEasy-1, even though the combined size of transgenes (~7 kb for Cbh-Cas9-mCherry and U6-sgRNA) is well below the tested size limit of 10 kb^21^. A small number of small plaques were formed but they failed to produce infectable adenoviral particles. We believe that the polarity of transgenes may affect the packaging process. For our new construct, specific sgRNA again is cloned by two rounds of PCR followed by LR reaction with 100% efficiency (Fig. 4B). The *Bmal1*-exon 6 targeting sgRNA was selected and cloned into the pAdTrack-Cas9-DEST, combined with pAdEasy-1, and then transfected into the packaging cell line 293 A.

We targeted *Bmal1* exon 6 because our previous transfection experiments found that targeting exon 6 produced more arrhythmic clones (62%) than targeting other *Bmal1* exons. Considering significant wt contamination (15–30%), targeting near this specific splicing site (Fig. 2A) probably produced frame-shifting mutations at a higher rate than the 2/3 rate expected based on random indels. The all-in-one adenovirus targeting *Bmal1* exon 6 was highly effective in infecting U2OS cells with close to 100% transduction efficiency (Fig. 4C, Supplementary Fig. 5). The measured frequency of indels was ~70% based on T7E1 assay with the genomic DNA extracted from the infected cells (Fig. 4D). However, since T7E1 and Surveyor enzymes do not cut effectively single-base mismatches or small mismatches, the actual indel frequency could be higher^40,41^. Indeed, immunoblotting showed that endogenous BMAL1 was hardly detectible in the treated cells (Fig. 4E). Accordingly, bioluminescence rhythms were not detectable when the transduced cells were set up for bioluminescence assay without clonal separation (Fig. 4F). To demonstrate that our all-in-one AV can achieve similarly high knockout efficiency on a different gene, an effective target was selected in *Per1* exon 6 (Supplementary Fig. 6) from a screening of several targets by transfection followed by T7E1 assay, and was knocked out using the all-in-one AV-*Per1*-E6 in U2OS cells. When U2OS cells were treated at MOI of 50 repeated twice, as described above, PER1 protein was barely detectible in the cells (Supplementary Fig. 6), confirming high knockout efficiency.

## Discussion

As with most biological fields, our current understanding of the circadian clock has been shaped by genetic manipulations that have led to identification of a dozen essential clock genes to date^22^. Traditional gene targeting using the homologous recombination (HR) machinery was revolutionary in editing the genome to produce precisely engineered knockout and knockin mice. The functional insights from these genetically engineered mouse models cannot be overstated. However, it is also important to recognize the mechanistic insights gained by studying mutant (and wt) cells in culture, as the clock mechanism is cell-autonomous and largely conserved across cell types^26,39,42^. Cell culture models will continue to be critically important for future advances in circadian biology, given their time- and cost-efficiencies. As our understanding of a biological pathway is most significantly advanced by identification of relevant new genes, a next frontier in circadian biology would be to identify new clock genes. We also have much to learn still from the hundreds of natural polymorphisms in known clock genes that lack a corresponding animal model. It has been already demonstrated that some of these polymorphisms are associated with circadian disorders such as Familial Advanced Sleep Phase Syndrome^43,44^. In the pre-CRISPR era, mutant cells were usually derived from mutant animals, as cell genomes could not be easily modified in culture. Now, however, cell culture models can be precisely edited using CRISPR-Cas9.

As demonstrated in this study, the phenotype of a mutant gene can be studied in cell models by transfection of an all-in-one CRISPR-Cas9 plasmid followed by clonal selection and expansion. Generation of the all-in-one plasmid has been simplified by using the Gateway method. However, the requirement for clonal selection/expansion is cumbersome, prone to contamination (In our hands, it was not possible to trypsinize U2OS cells into single cells with ~100% efficiency, which made FACS-sorted cells contaminated with non-transfected cells.), and not possible for all cell types. We show that CRISPR-Cas9 combined with AV can remove the need for clonal selection and expansion, thus addressing all of those issues in AV-infectible cells (Fig. 4G). We and others have shown that AV can infect diverse primary and immortalized cells and transduce transgenes with ~100% efficiency. Some cell lines such as MEFs require higher titers of adenovirus to achieve 100% transduction compared to other cell lines such as U2OS^19^, but adenovirus can be easily concentrated by density gradient ultracentrifugation to achieve higher titers^21^. Adenoviral titer can be adjusted to maximize infection rate without cytotoxicity by visual inspection of fluorescence and cell morphology. We routinely measure robust bioluminescence rhythms from ~100% AV-infected MEFs and U2OS cells for several days, demonstrating that the physiology of these cells are not compromised^19,45^. Since AV transduction can be 100% efficient in delivering Cas9:sgRNA, whereas FACS sorting following transfection still includes non-transfected cells, AV is a more efficient way to achieve knockouts even when FACS sorting is feasible. A simple serial dilution without FACS sorting would be enough for clonal isolation and expansion for downstream assays (Fig. 4G).

Even if transduction of Cas9-sgRNA is near 100% and if Cas9-sgRNA can induce indels in ~100% of cells, one may expect frame-shifting mutations to occur only in 2/3 of cases. However, based on our results, the NHEJ repair process is not random, but rather locus-specific (e.g., compare our sequencing results between *Bmal1*-E7 and *Per2*-E15 in Fig. 2C and Supplementary Fig. 1). Further, targeting loci near splicing sites can increase the chance of null mutations by generating alternative nonfunctional splicing variants as well as frame-shifting mutations, as is the case for targeting of *Bmal1*-E6. As a future refinement, we propose that the efficiency can be further increased by using two different adenoviruses targeting two different early exons. We have shown that MEFs could be doubly transduced by AV^26^. Although AV has been tested to deliver Cas9 and/or sgRNA into cells and showed knockdown of target proteins up to 90% in some cases^15^, to our knowledge, our present study is the first one to demonstrate a functional knockout without clonal selection by an all-in-one AV. All AV viral genes can be removed in the AV vector to increase packaging capacity and reduce immune response *in vivo*^14^. However, when the AV vector devoid of all viral genes is used, packaging efficiency suffers, resulting in vector titers ~two orders of magnitude lower, even after multiple rounds of amplification, compared to ours^14^. AV can also be employed to deliver catalytically-inactive Cas9 as a synthetic transcription factor. It has been shown that a catalytically-inactive Cas9 can be targeted to promoters to activate or inhibit transcription of their downstream genes^10^. AV is superior to lentivirus for that purpose because expression levels of transgenes are much higher by AV than lentivirus.

In summary, our all-in-one AV-CRISPR tool both simplifies the sgRNA cloning step and eliminates clonal selection and expansion in adenovirus-infectible cells, which allows small laboratories to generate mutant cell lines easily in a rapid manner without extensive knowledge in cell culture or access to a FACS machine (Fig. 4G). Moreover, this streamlined approach is amenable to high-throughput screening: at the cloning step, selection of positive Cas9-sgRNA recombinants is not required when a library of Entry PCR amplicons is recombined with the all-in-one plasmids by LR clonase.

## Materials and Methods

### Plasmid construction

pEntry-sgRNA was generated by Mutagenex (530 Highland Station Dr., Suwanee, GA) as follows. U6-sgRNA fragment from pU6-(BbsI)_Cbh-Cas9-T2A-mCherry plasmid (Addgene #64324^46^) was PCR-amplified with primers with attL1 and attL2 sequence and subcloned into pEAR A1A (Supplementary Fig. 9).

pShuttle-Cas9-DEST plasmid was constructed by two steps of subcloning. First, Cbh-Cas9-T2A-mCherry-polyA fragment from the pU6-(BbsI)_Cbh-Cas9-T2A-mCherry plasmid (Addgene #64324) was subcloned into XbaI and NotI sites of pShuttle (Addgene #16402^21^). Then, R1-Cm^R^-ccdB-R2 fragment from the Gateway pDEST (ThermoFisher Scientific, Hampton, NH) was subcloned into the intermediate vector (Supplementary Fig. 10).

pAdTrack-Cas9-Dest was constructed by subcloning Cbh-Cas9 and polyA fragments from the pU6-(BbsI)_Cbh-Cas9-T2A-mCherry plasmid (Addgene #64324), and R1-Cm^R^-ccdB-R2 fragment from the Gateway pDEST into KpnI/HidIII, HindIII and KpnI sites of pAdTrack (Addgene #16404^21^), respectively (Supplementary Fig. 11).

### Generation of pEntry-sgRNA amplicons by two step PCR and LR cloning

The first round of PCR was performed using two sets of primers as described in Table 1. The second round of PCR was performed using a mixture of two PCR products (1 ul each of 100-fold diluted PCR product) as templates and L1 + L2 primers (Table 1). The same thermal cycling conditions were used. Note that extra c and g (lower case) were appended to U6-Rev and Scaff-fwd primers to allow the sgRNA to start with “G” if the target seq does not start with “G” as recommended by Ran *et al*.^4^ The final PCR products were purified and mixed with either pShuttle-Cas9-DEST or pAdTrack-Cas9-DEST along with LR clonase (Thermo Fisher Scientific #11791020) to generate final all-in-one vectors. Bacterial colonies transformed with the reaction mixture were screened by colony PCR that amplifies DNA region flanked by R1 and R2 (negative clones), or B1 and B2 (positive recombinants). Two different sets of primers were used for pShuttle and pAdTrack as follows.

**Table 1:** PCR primers and condition for generating pEntry-sgRNA.

	PCR primer used	PCR product size	Thermal cycling condition
PCR reaction (1) PCR reaction (2) PCR reaction (3)	L1 + U6-rev Scaff-fwd + L2 L1 + L2	475 bp 398 bp 824 bp	1. 95 °C 5 min 2. 95 °C 20 sec 3. 62 °C 20 sec 4. 72 °C 20 sec 5. 2- > 4 repeat 30 times 6. 72 °C 5 min 7. 4 °C hold
Primer sequence	L1:5′-GGCTGCAGGAATTCGATAAAAAGCTC-3′ L2:5′-CCCAGTCACGACGTTGTAAAACGACG-3′ U6-Rev:5′-TTCTAGCTCTAAAACNNNNNNNNNNNNNNNNNNNN cGGTGTTTCGTCCTTTCCACAAG-3′ U6-scaff:5′-AGGACGAAACACCgNNNNNNNNNNNNNNNNNNNN GTTTTAGAGCTAGAAATAGCAAG-3′		

pShuttle-Cas9-DEST:

attR1 Up Fwd: 5′-GAGCCCACTGCTTACTGGCTTATC-3′

Cbh Rev: 5′-CGTACTTGGCATATGATACACTTGA-3′

Size of PCR amplicons: 1,966 bp negative clone and 691 bp for positive recombinant.

pAdTrack-Cas9-DEST:

KpnIattR1F: 5′-ATAGGTACCCCCACTGCTTACTGGCTTATCGAAATTAATAC-3′

KpnIattR2R: 5′-CCGTAAGTTATGTAACGGGTACCTCTAGATCAACCAC-3′

Size of PCR amplicons: 1820 bp for negative clone and 545 bp for positive recombinant.

### Transfection and T7E1 assay

A U2OS cell line expressing luciferase under a *Bmal1* promoter was described previously. For transfection with the all-in-one plasmids, cells were plated into 6 cm dishes to be approximately 60% confluent on the day of transfection. Cells were transfected with Polyfect (Qiagen, Hilden, Germany) according to the manufacturer’s protocol and incubated for 2 days before they were subjected to trypsinization and FACS sorting using BD FACSAria SORP equipped with an Automated Cell Deposition Unit (ACDU) for sorting into 96 well plates. Cells were trypsinized with Trypsin (0.25%)-EDTA (2.21 mM) for 3 min and were filtered through mesh with 50um pores. FACS-sorted cells were collected into three groups: negative, intermediate and bright mCherry, and plated into 35 mm dishes and grown for 2 days before harvest and genomic DNA extraction. Before the cells were harvested, they were inspected for mCherry expression. Even the bright mCherry group included 10–30% of non-mCherry cells. Harvested cell pellets were homogenized in 250 ul solution A containing 0.1 M Tris-HCl pH = 9.0, 0.1 M EDTA, and 1% SDS, and incubated at 70 °C for 30 minutes. 35 ul 8 M potassium acetate was added and incubated at room temperature for 5 minutes. The samples were centrifuged at 13,000 rpm for 15 minutes and genomic DNA was purified by subjecting the supernatant to phenol-chloroform extraction and ethanol precipitation. The extracted genomic DNA pellet was dissolved in 100 ul water and used as a template to PCR amplify the target genomic locus with a set of primers (Table 2) flanking the target region. Amplicons were confirmed by agarose electrophoresis, and purified with PCR purification kit (QIAGEN Cat.28104). 200 ng DNA samples were denatured and annealed according to the protocol in Ran *et al*. and subjected to T7E1 digestion in a 20 ul reaction according to the manufacturer’s protocol (NEB cat.M0302). Digestion products were resolved on 8% acrylamide/bis TBE gel and visualized with EtBr.

**Table 2:** PCR primers for T7E1 assay.

Targeted gene & region	T7E1 PCR primer left	T7E1 PCR primer right	Anticipated PCR product size	Anticipated cleavage product size
Bmal1 exon 6	CTGTGGCTGTTCGAACTTTATG	ACATTGCTGTTTTCTTCTGCCT	378 bp	159 bp, 219 bp
Bmal1 exon 7	CTCAACTGGAGATGAGCAAGG	GCCTTGATTGATTTCTGCTACC	485 bp	179 bp, 306 bp
Bmal1 exon 8	TGAGAGGAAAAGAAACGAGGAG	TGAGAGGAAAAGAAACGAGGAG	468 bp	148 bp, 320 bp
Bmal1 exon 9	CCATGGAATTCTCTTTGGCTTA	AGGAGAATGGTTTTGTGGAAGA	461 bp	195 bp, 266 bp
PER2 exon 5	GAACTCTGCGACCGTATTACCT	CTTATCTGCTGAAACCCCAAAC	519 bp	174 bp, 345 bp
PER2 exon 8_1	GGTGTTAACTCTGATTTGCCCT	GTGTGCTGAGTCTCCAGAAAGA	513 bp	200 bp, 313 bp
PER2 exon 8_2	CAGAGCAGAGGTACACATCACC	ACTTGTCAGAGCTGTTCCCACT	580 bp	159 bp, 421 bp
PER2 exon 15	GAGCATAAAGTACGTGGGCTCT	TACCTGTGTGAAAGGCATGAAC	554 bp	166 bp, 388 bp
PER1 exon 6	GTAAGTGGTGTGTCCCAAGAGGGA	CTAGGCTGCGAAGAATCCACTAAG	615 bp	402 bp, 213 bp

### TOPO-PCR cloning and sequencing

PCR amplicons obtained above were cloned into plasmids using the TOPO-PCR cloning kit (Invitrogen #K2800), and inserts were sequenced from 20 colonies each for *Bmal1* exon 7 and *Per2* exon 15. Wt contamination was confirmed: 4 out of 16 were wt for *Bmal1* exon 6 and 2 out of 15 were wt for *Per2*.

### Single cell isolation and expansion, bioluminescence monitoring and immunoblotting

mCherry-expressing cells were singly sorted into 96 well plates by FACS and expanded serially into 24 well plates, 6 well plates, and then 10 cm dishes. A few dozen clones for each of *Bmal1* exon 6, 8 and 9, and *Per2* exon 5 were set up for bioluminescence monitoring as described previously^19^. For immunoblotting for BMAL1, a previously described anti-BMAL1 antibody (GP3) was used^47^. For human PER2, novel polyclonal anti human PER2 antibodies were generated in guinea pigs using aa 1–200 peptide by Cocalico Biologicals, Inc (449 Stevens Rd, Reamstown, PA). hP2-GP49 was used in the current study. The antibody was validated by oscillations of human PER2 in U2OS cells and a novel monoclonal antibody against human PER2 (hP2-C6A3). The monoclonal antibody was selected from clones of antibodies raised against aa 1–200 of human PER2 by Boreda Biotech (Seongnam, Gyeonggi-Do, Korea). The monoclonal antibody was also able to detect oscillations of PERs in U2OS cells similarly (Supplementary Fig. 7). Uncropped immunoblot images are shown in Supplementary Fig. 12.

### Generation of adenovirus

Adenoviruses expressing mutant BMAL1 lacking the DNA-binding domain or wt BMAL1 were described previously^19,38^. To generate adenovirus expressing Cas9 and sgRNA targeting *Bmal1* exon 6, pAdTrack-Cas9-U6-Bmal1E6 sgRNA plasmid was used to transform electrocompetent BJ5183 cells harboring pAdEasy1 by electroporation. Positive recombinants were selected by PacI digestion and transfected into the packaging cell line 293A in a T25 flask. Cells were harvested along with the medium 10–12 days later. Adenovirus was released from the cells by freezing and thawing cells + medium three times and harvested by centrifugation. The supernatant was used to infect confluent 293 cells in two T75 flasks. Final adenoviral prep was obtained by 3 cycles of freezing-thawing cell + medium from two T75 flasks and centrifugation. The final lysate contains ~ 3 × 10^7^ transducing units (TU). When compared with 293 cells, expression forming units (efu) of the adenovirus was 5–10 fold lower for U2OS cells. However, U2OS cells could be infected with ~100% efficiency without further purification, unlike infection for MEFs^19^. To ensure 100% infection in U2OS cells with the all-in-one adenovirus, the cells were infected at MOI of 50 twice, two days apart.

### Full sequence of pEAR-A1A plasmid

#### > pEAR-A1, Mutagenex ver. 08/0315, 2604 bp, Amp^R^

CACCTGACGCGCCCTGTAGCGGCGCATTAAGCGCGGCGGGTGTGGTGGTTACGCGCAGCGTGACCGCTACACTTGCC

AGCGCCCTAGCGCCCGCTCCTTTCGCTTTCTTCCCTTCCTTTCTCGCCACGTTCGCCGGCTTTCCCCGTCAAGCTCT

AAATCGGGGGCTCCCTTTAGGGTTCCGATTTAGTGCTTTACGGCACCTCGACCCCAAAAAACTTGATTAGGGTGATG

GTTCACGTAGTGGGCCATCGCCCTGATAGACGGTTTTTCGCCCTTTGACGTTGGAGTCCACGTTCTTTAATAGTGGA

CTCTTGTTCCAAACTGGAACAACACTCAACCCTATCTCGGTCTATTCTTTTGATTTATAAGGGATTTTGCCGATTTC

GGCCTATTGGTTAAAAAATGAGCTGATTTAACAAAAATTTAACGCGAATTTTAACAAAATATTAACGCTTACAATTT

CCATTCGCCATTCAGGCTGCGCAACTGTTGGGAAGGGCGATCGGTGCGGGCCTCTTCGCTATTACGCCAGCTGGCGA

AAGGGGGATGTGCTGCAAGGCGATTAAGTTGGGTAACGCCAGGGTTTTCCCAGTCACGACGTTGTAAAACGACGGCC

AGTGAATTGTAATACGACTCACTATAGGGCGAATTGGGTACCGGGCCCCCCCTCGAGGTCGACGGTATCGATAAGCT

TGATATCGAATTCCTGCAGCCCGGGGGATCCACTAGTTCTAGAGCGGCCGCCACCGCGGTGGAGCTCCAGCTTTTGT

TCCCTTTAGTGAGGGTTAATTTCGAGCTTGGCGTAATCATGGTCATATGCCATGGCCAGGAACCGTAAAAAGGCCGC

GTTGCTGGCGTTTTTCCATAGGCTCCGCCCCCCTGACGAGCATCACAAAAATCGACGCTCAAGTCAGAGGTGGCGAA

ACCCGACAGGACTATAAAGATACCAGGCGTTTCCCCCTGGAAGCTCCCTCGTGCGCTCTCCTGTTCCGACCCTGCCG

CTTACCGGATACCTGTCCGCCTTTCTCCCTTCGGGAAGCGTGGCGCTTTCTCATAGCTCACGCTGTAGGTATCTCAG

TTCGGTGTAGGTCGTTCGCTCCAAGCTGGGCTGTGTGCACGAACCCCCCGTTCAGCCCGACCGCTGCGCCTTATCCG

GTAACTATCGTCTTGAGTCCAACCCGGTAAGACACGACTTATCGCCACTGGCAGCAGCCACTGGTAACAGGATTAGC

AGAGCGAGGTATGTAGGCGGTGCTACAGAGTTCTTGAAGTGGTGGCCTAACTACGGCTACACTAGAAGGACAGTATT

TGGTATCTGCGCTCTGCTGAAGCCAGTTACCTTCGGAAAAAGAGTTGGTAGCTCTTGATCCGGCAAACAAACCACCG

CTGGTAGCGGTGGTTTTTTTGTTTGCAAGCAGCAGATTACGCGCAGAAAAAAAGGATCTCAAGAAGATCCTTTGATC

TTTTCTACGGGGTCTGACGCTCAGTGGAACGAAAACTCACGTTAAGGGATTTTGGTCATGAGATTATCAAAAAGGAT

CTTCACCTAGATCCTTTTAAATTAAAAATGAAGTTTTAAATCAATCTAAAGTATATATGAGTAAACTTGGTCTGACA

GTTACCAATGCTTAATCAGTGAGGCACCTATCTCAGCGATCTGTCTATTTCGTTCATCCATAGTTGCCTGACTCCCC

GTCGTGTAGATAACTACGATACGGGAGGGCTTACCATCTGGCCCCAGTGCTGCAATGATACCGCGAGAACCACGCTC

ACCGGCTCCAGATTTATCAGCAATAAACCAGCCAGCCGGAAGGGCCGAGCGCAGAAGTGGTCCTGCAACTTTATCCG

CCTCCATCCAGTCTATTAATTGTTGCCGGGAAGCTAGAGTAAGTAGTTCGCCAGTTAATAGTTTGCGCAACGTTGTT

GCCATTGCTACAGGCATCGTGGTGTCACGCTCGTCGTTTGGTATGGCTTCATTCAGCTCCGGTTCCCAACGATCAAG

GCGAGTTACATGATCCCCCATGTTGTGCAAAAAAGCGGTTAGCTCCTTCGGTCCTCCGATCGTTGTCAGAAGTAAGT

TGGCCGCAGTGTTATCACTCATGGTTATGGCAGCACTGCATAATTCTCTTACTGTCATGCCATCCGTAAGATGCTTT

TCTGTGACTGGTGAGTACTCAACCAAGTCATTCTGAGAATAGTGTATGCGGCGACCGAGTTGCTCTTGCCCGGCGTC

AATACGGGATAATACCGCGCCACATAGCAGAACTTTAAAAGTGCTCATCATTGGAAAACGTTCTTCGGGGCGAAAAC

TCTCAAGGATCTTACCGCTGTTGAGATCCAGTTCGATGTAACCCACTCGTGCACCCAACTGATCTTCAGCATCTTTT

ACTTTCACCAGCGTTTCTGGGTGAGCAAAAACAGGAAGGCAAAATGCCGCAAAAAAGGGAATAAGGGCGACACGGAA

ATGTTGAATACTCATACTCTTCCTTTTTCAATATTATTGAAGCATTTATCAGGGTTATTGTCTCATGAGCGGATACA

TATTTGAATGTATTTAGAAAAATAAACAAATAGGGGTTCCGCGCACATTTCCCCGAAAAGTGC

NNN: MCS

T7-F 5′-TAATACGACTCACTATAGGG

T3-F 5′-ATTAACCCTCACTAAAGGG

A1 Seq-F 5′-GATTAAGTTGGGTAACGCCAGGG

A1 Seq-R 5′-CTTGAGCGTCGATTTTTGTGATGC

A1 Flk-F 5′-GTAATACGACTCACTATAGGGC.

## Electronic supplementary material

Supplementary figures
