# Supplementary material for: Streamlined procedure for gene knockouts using all-in-one adenoviral CRISPR-Cas9
Source: Sci Rep. 2019 Jan 22;9:277. doi: 10.1038/s41598-018-36736-y (PMC6342919; doi:10.1038/s41598-018-36736-y)
Supplement: Supplementary file 1 — Supplementary figures [file 41598_2018_36736_MOESM1_ESM.docx]

Supplementary Information for

Streamlined procedure for gene knockouts using all-in-one adenoviral CRISPR-Cas9

Yuan-Hu Jin^a^, Hyunjeong Joo^a,b^, Kwangjun Lee^a^, Hyeongseok Kim^c^ ,Ruth Didier^a^, Young Yang^b^, Heungsop Shin^a,c,1^, Choogon Lee^a,1^

Heungsop Shin

Email: hshin@kpu.ac.kr

Choogon Lee

Email: [Choogon.lee@med.fsu.edu](mailto:Choogon.lee@med.fsu.edu)

**This PDF file includes:**

Figs. S1 to S12

References for SI reference citations


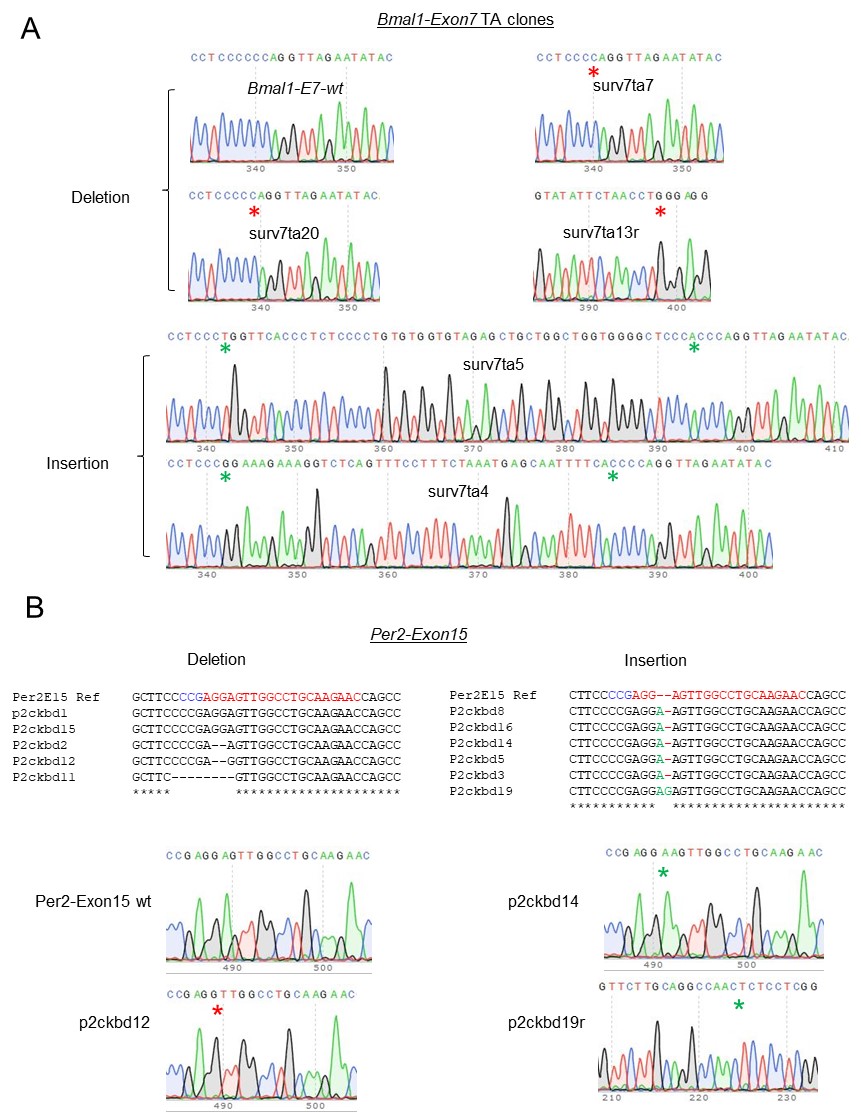


**Supplementary Figure 1**. **Diverse indels are generated in *Bmal1* and *Per2* genes by the all-in-one Cas9-sgRNA.**

(A) Raw sequencing data of PCR amplicons from *Bmal1* exon 7 mutated by the all-in-one Cas9-sgRNA. Red and green asterisks indicate deletions and insertions, respectively. (B) Indels for *Per2* exon 15 is shown after it is targeted by the all-in-one vector.


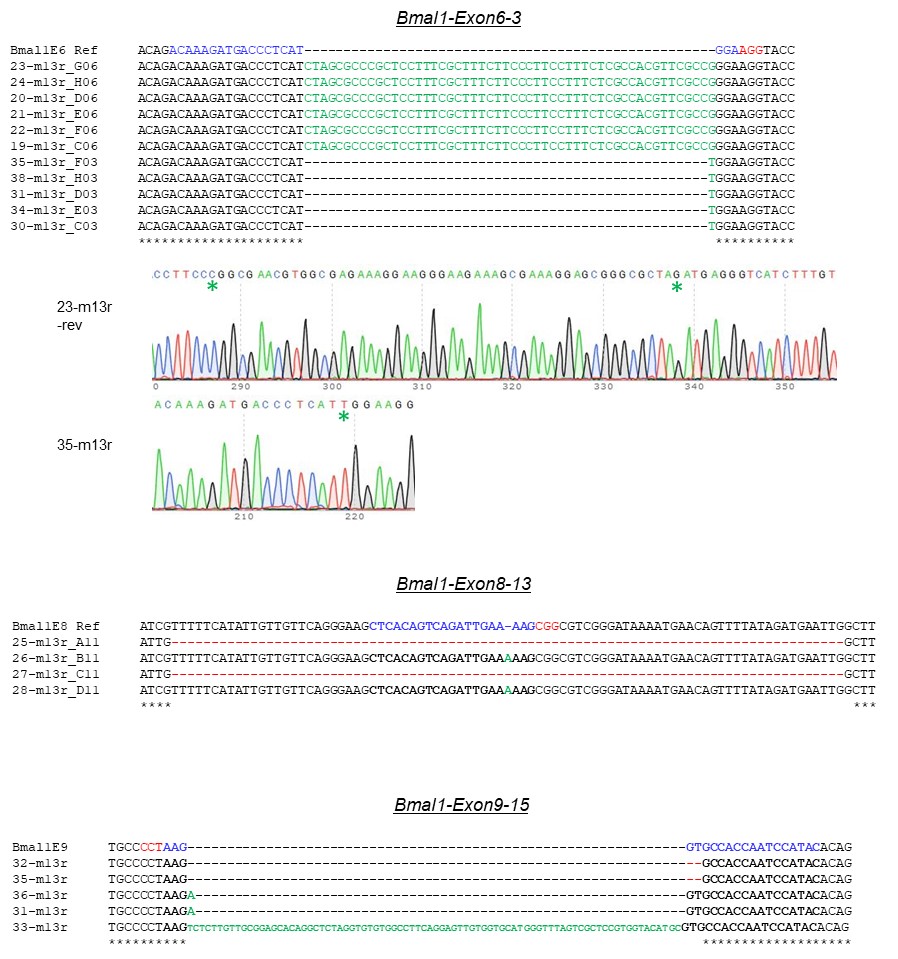


**Supplementary Figure 2**. **Frame-shifting mutations in *Bmal1* in a subset of arrhythmic clones are confirmed by sequencing.** A dozen clones from PCR amplicons were sequenced after they were cloned into a sequencing vector. Sequencing data of 11 clones from a PCR amplicon are shown for *Bmal1* exon 6-3. Note that there are two different insertions. Four clones are shown for *Bmal1* exon 8-13 and five clones for exon 9-15. Note that there are three different alleles for 9-15, suggesting that the original cell clone had at least three copies of the gene. It is known that U2OS cells often have aneuploidy^1^.

**
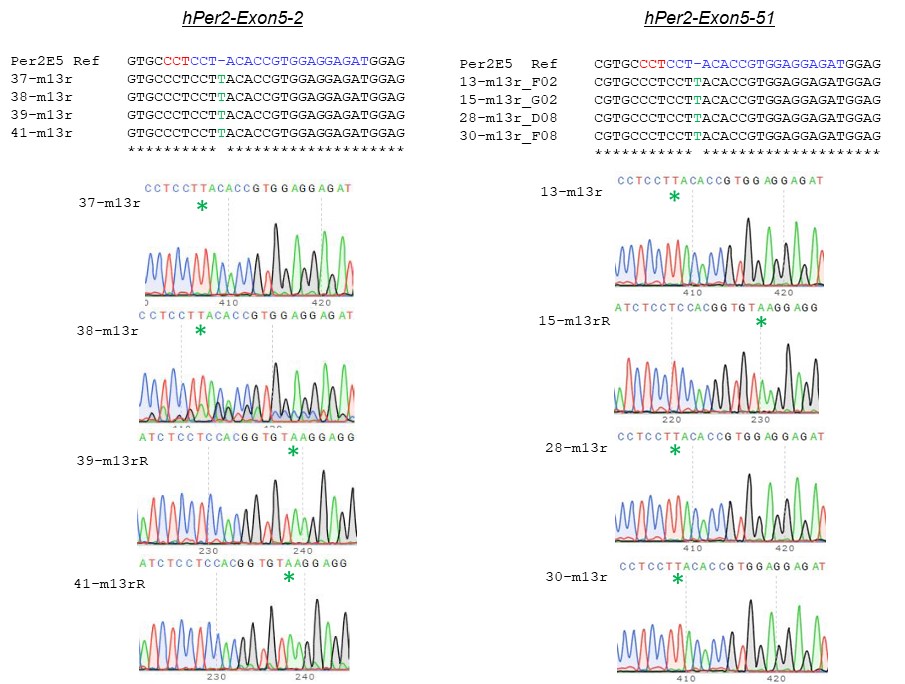
**

**Supplementary Figure 3. Frame-shifting mutations in *Per2* mutant clones are confirmed by sequencing.** PCR amplicons from two mutant *Per2* exon 5 cell clones were sequenced as above. In both cell clones, there was one “T” insertion in all alleles based on sequencing of 12 PCR clones each.


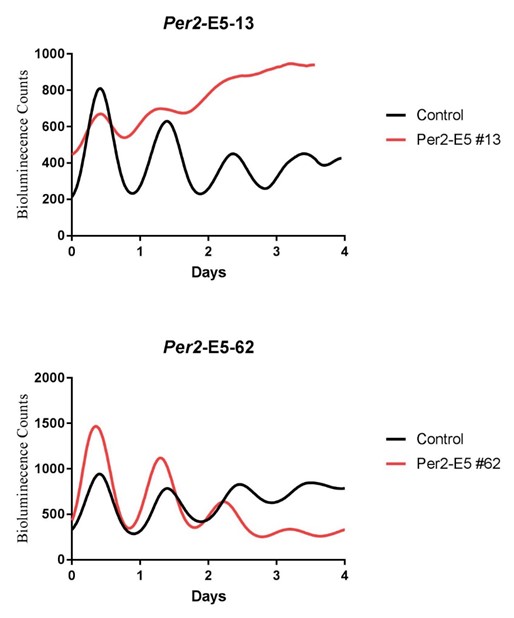


**Supplementary Figure 4. Non-frame-shifting mutations generated in the *Per2* gene by the all-in-one vector result in disruption of circadian rhythms.** Two *Per2* mutant clones showed significantly disrupted rhythms, but retained normal levels of expression of PER2. Both period (hr) and amplitude (RU) are significantly different between mutant clone #5-13 and wt, but only period is different between #5-62 and wt.

Wt vs. #5-13 = 23.4+/-0.1 vs. 21.7+/-0.4 hr (p<0.01); 221.3+/-30.5 vs. 55.6+/-15.4 RU (p<0.01).

Wt vs. #5-62 = 24.2+/-0.3 vs. 22.1+/-0.2 hr (p<0.001); 226.2+/-31.1 vs. 192.4+/-61.3 RU (p>0.05).

Values are mean+/-SD, from 3 samples per group, compared using unpaired t-test.


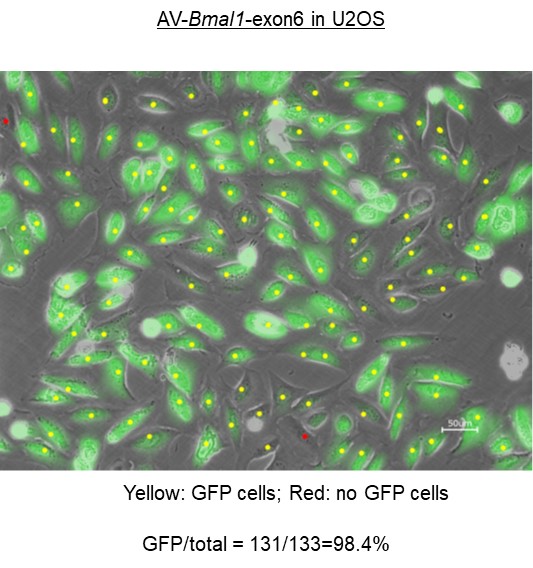


**Supplementary Figure 5. High transduction efficiency of AV.** U2OS cells were transduced with all-in-one AV-*Bmal1*-exon 6 twice at MOI of 50. Total cells include GFP + non GFP cells.


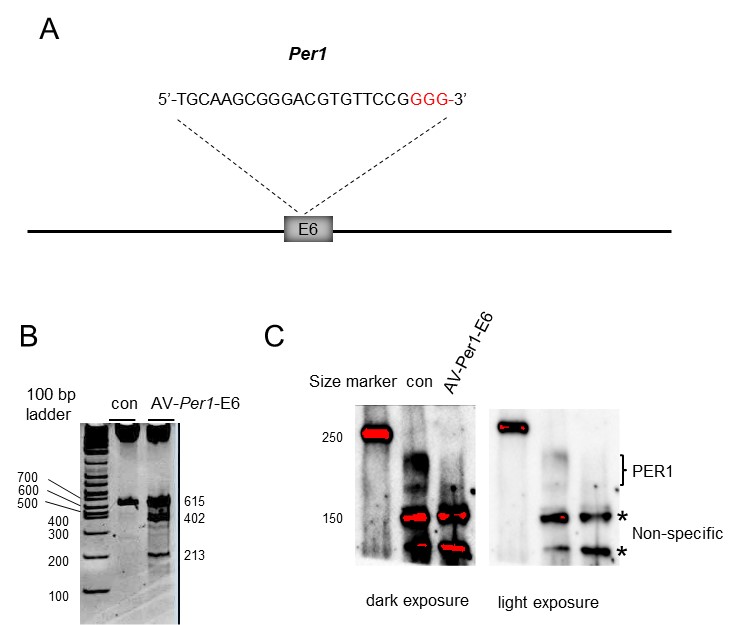


**Supplementary Figure 6. Effective knockdown of PER1 protein in heterogenic U2OS cells by all-in-one AV-*Per1*-exon 6.** (A) Target sequence is shown along with PAM (red). (B) T7E1 assay after double transduction with all-in-one AV-*Per1*-exon6. (C) Immunoblot for endogenous PER1. Two exposures are shown.


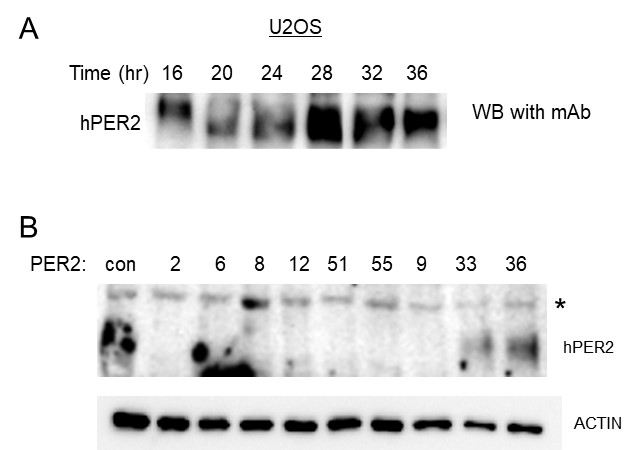


**Supplementary Figure 7. Novel anti-human PER2 polyclonal (hP2-GP49) and monoclonal (hP2-C6A3) antibodies detect the endogenous protein in U2OS cells.** (A) Immunoblotting of U2OS time course samples with hP2-C6A3. The samples were harvested at the indicated times after a 2-hr serum shock. (B) *Per2* exon 5 cell clones were harvested at a different time than the one in Fig. 3 and subjected to immunoblotting with hP2-GP49.


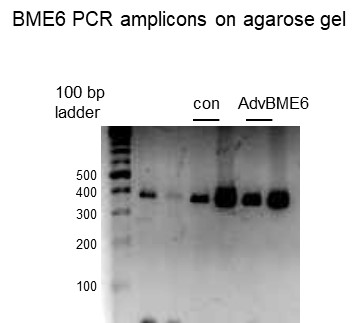


**Supplementary Figure 8. BME6 amplicons on agarose gel.** BME6 amplicons run as expected on agarose gel.


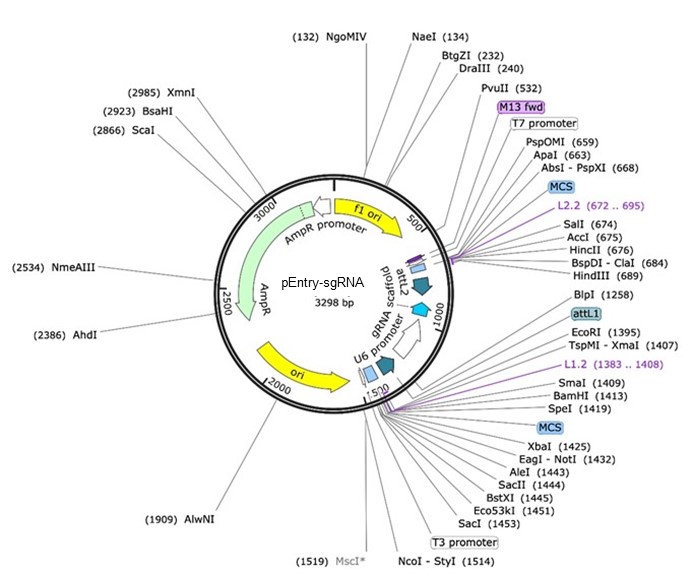


**Supplementary Figure 9. Map of pEntry-sgRNA.** This plasmid was used as the template to generate specific sgRNA amplicons.


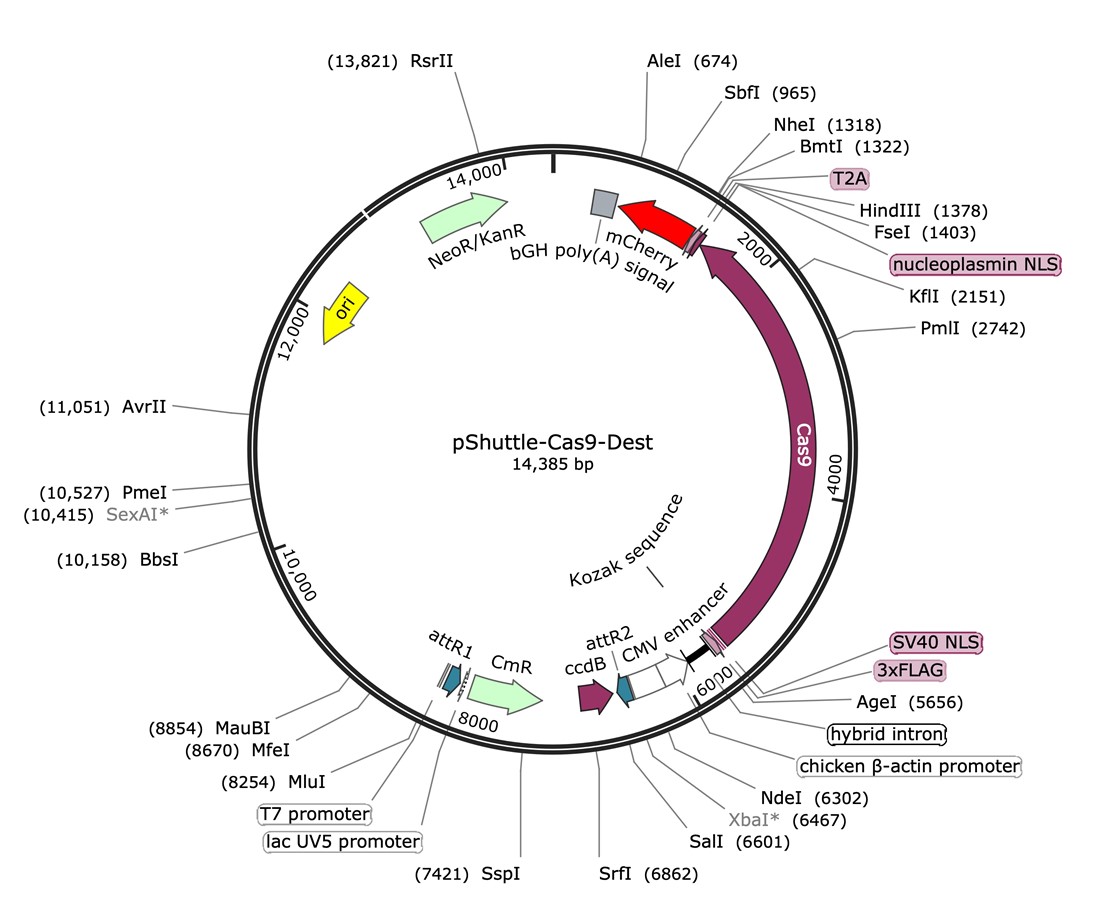


**Supplementary Figure 10. Map of pShuttle-Cas9-Dest.** Note that Cas9-mCherry fusion protein is expressed under the single Cbh promoter.


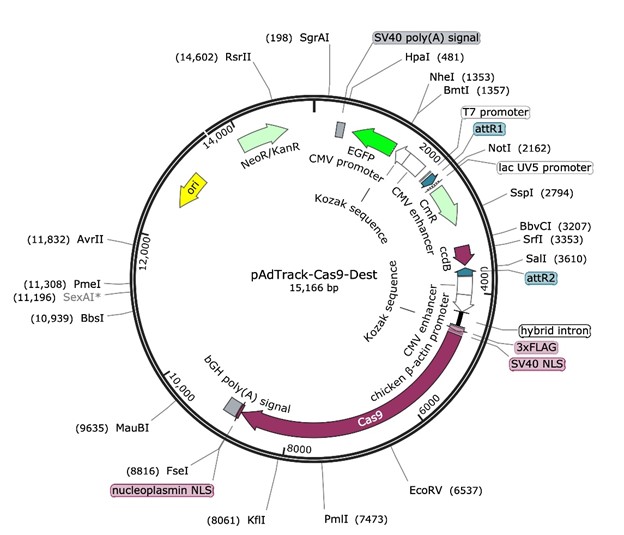


**Supplementary Figure 11. Map of pAdTrack-Cas9-Dest.** Note that EGFP and Cas9 are expressed by two separate CMV and Cbh promoters, respectively.


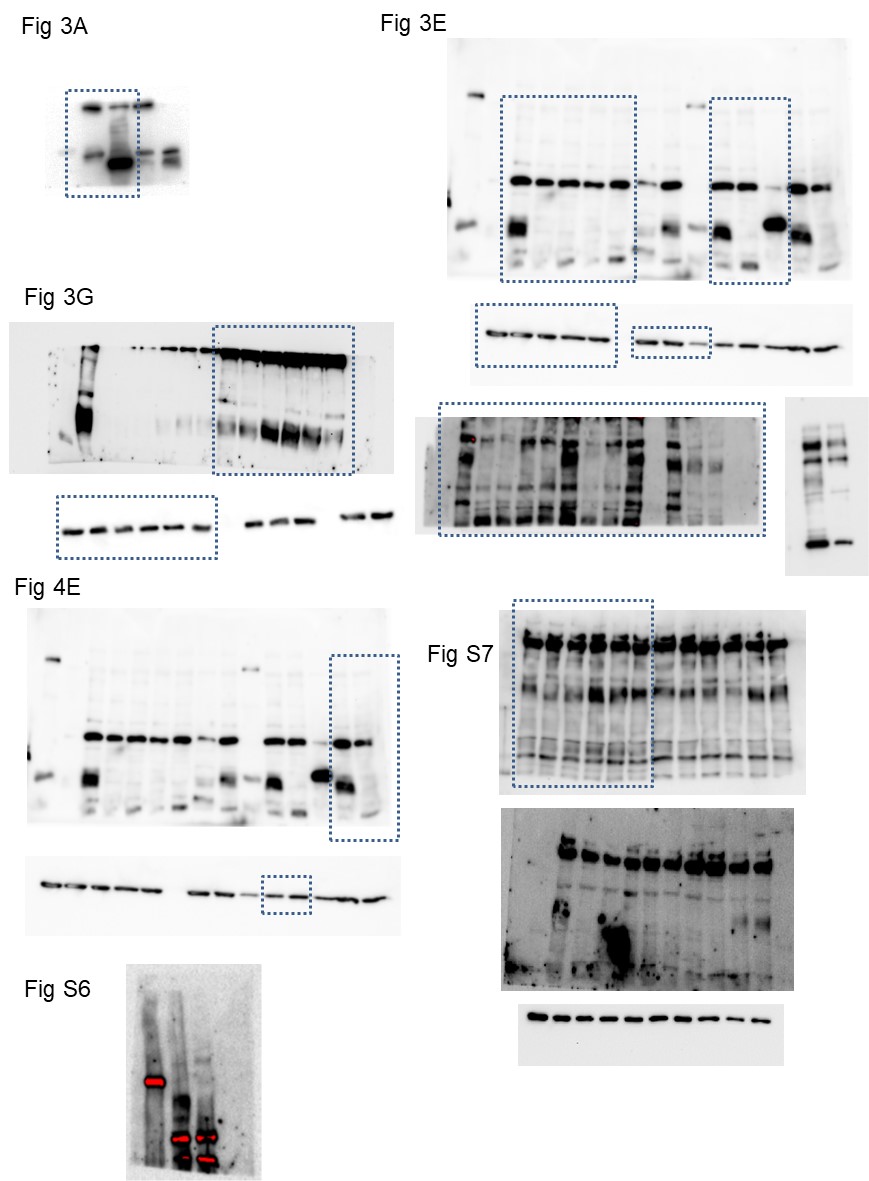


**Supplementary Figure 12. Uncropped images of the blots presented in the main text.** Blue boxes indicate the portions of images used in the main text.

References

1. Ozaki T*, et al.* (2003) Chromosomal alterations in osteosarcoma cell lines revealed by comparative genomic hybridization and multicolor karyotyping. *Cancer Genet Cytogenet* 140(2):145-152.
